# DNA Barcoding of Andaliman (*Zanthoxylum acanthopodium* DC) from North Sumatra Province of Indonesia Using *Maturase K* Gene

**DOI:** 10.21315/tlsr2021.32.2.2

**Published:** 2021-06-29

**Authors:** Cicik Suriani, Eko Prasetya, Tri Harsono, Johannes Manurung, Hary Prakasa, Dina Handayani, Miftahul Jannah, Yuanita Rachmawati

**Affiliations:** 1 Department of Biology, Faculty of Mathematics and Natural Sciences, Universitas Negeri Medan, Jl. Willem Iskandar, Pasar V, Medan Estate, Medan, 20221, North Sumatra, Indonesia; 2 Department of Biology, Faculty of Sciences and Technology, Islamic University of Assyafiiyah, Jl. Jatiwaringin Raya, No 12, East Jakarta,17411 Indonesia; 3 Department of Biology, Faculty of Sciences and Technology, Islamic State University Sunan Ampel Surabaya, Jl. Ahmad Yani 117 Surabaya, East Java, 60237, Indonesia

**Keywords:** *Z. acanthopodium*, *Maturase K*, North Sumatra, DNA Barcoding

## Abstract

Andaliman (*Zanthoxylum acanthopodium* DC) is a native plant of North Sumatra province. *Zanthoxylum acanthopodium* is a member of *Rutaceae* family widely found in northern Sumatra, Indonesia. The aim of this study was to barcode *Z. acanthopodium* in North Sumatra province, Indonesia based on cpDNA *maturase K* (*matK*). Samples were collected in seven localities across six regions of North Sumatra province. Phylogenetic analysis was conducted using Maximum Likelihood method. The results of phylogenetic analysis indicate that *Z. acanthopodium* is a monophyletic group that is derived from a common ancestor. The results of the phylogenetic tree construction show that there is a grouping of accession between *Z. acanthopodium* species separate from other species in the *Zanthoxylum* genus as well as those of the Rutaceae family. The results showed that cpDNA *matK* marker can effectively be used as DNA barcoding to identify *Z. acanthopodium*.

**Highlights**

- cpDNA *maturase K* gene marker sequence can be used as DNA barcodes to identify *Zanthoxylum acanthopodium*.
- Based on the cpDNA *maturase K* gene sequence on *Zantoxylum acanthopodium* had very high homology (97.2%).
- The genetic distance between *Zanthoxylum acanthopodium* using the cpDNA *maturase K* gene sequence is very small (<1%).

## INTRODUCTION

Andaliman (*Zanthoxylum acanthopodium* DC) is a native plant of North Sumatra province in Indonesia (Suryanto *et al*. 2004). *Zanthoxylum acanthopodium* is a member of the Rutaceae family that is widely spread throughout the northern part of Sumatra, Indonesia (Siregar 2003). This fruit is commonly used as spice in traditional Batak cuisine (Kristanty & Suriawati 2015). Spice made of *Z. acanthopodium* is known by the name of “batak pepper” since it is pominent in traditional Batak cuisine (Hidayah 2015).

*Zanthoxylum* is a member of the Rutaceae (Pirani 1993) family which consists of around 200 species spread across the region of Central Asia and North America (Hartley 1966) with natural characteristics of being thorny bushes, small trees with bushy and branched leaves and thorny stems, producing edible fruits with strong-smelling leaves (Chyau *et al*. 1996). *Z. acanthopodium* is known for its distinct cistrus-like flavour and spiciness with unique taste, rendering numbness and sharp pain on one’s tongue (Wijaya 2000). This plant has also been used to preserve foods such as raw fish and tofu (Parhusip *et al*. 1999).

*Zanthoxylum* is a complex genus with many different species and is not well-studied (Arun & Paridhavi 2012). Species in the *Zanthoxylum* genus have many similarities in its visible morphological characters, making it difficult to distinguish species within this genus (Gupta & Mandi 2013). Therefore, DNA sequence analysis is required to identify species within the *Zanthoxylum* genus. Research using molecular markers was performed to facilitate identification using morphological markers due to its higher stability (Yunus 2007) and resistance to environmental differences and robust plant life, resulting in more accurate data (Julisaniah *et al*. 2008).

Research on identification of *Z. acanthopodium* using DNA barcoding from chloroplast DNA (cpDNA) is still rare. Feng *et al*. (2015) analysed genetic variations and relationships between species in the *Zanthoxylum* genus using sequence-related amplified polymorphism (SRAP) marker. Putri *et al*. (2016) and Sembiring *et al*. (2015) analysed the genetic diversity of *Z. acanthopodium* using the Random Amplified Polymorphic DNA (RAPD) marker. Gupta and Mandi (2013) established DNA fingerprints using Amplified Fragment Length Polymorphism (AFLP) markers to differentiate *Z. acanthopodium* from *Z. oxyphyllum*.

Chloroplast DNA can be used to reveal diversity and trace evolutionary family tree of *Z. acanthopodium*. Chloroplast DNA has been widely used for phylogenic studies of various plants such as *Bouea* (Harsono *et al*. 2017), Sedoideae subfamily (Lim & Choi 2018), *Solanum* (Olmstead & Palmer 1997), and Aurantioideae subfamily (Bayer *et al*. 2009). Chloroplast DNA is used as a barcoding because it is easy to purify with a very conservative character and low evolution rate, so it can be used for philogenic analysis between taxa in flowering plant families (Clegg & Durbin 1990; Tsumura *et al*. 1996).

Chloroplast DNA is a double-chain DNA of circular shape (Didriksen 2010) which consists of various genes such as *rbcl, trnL-F* and *matK* (Kress & Erickson 2007; Kalangi *et al*. 2014). The Consortium for the Barcode of Life (CBOL) recommended *rbcL* and *matK* as the standard barcode (Hollingworth *et al*. 2009). The *matK* gene is more commonly used in various researches compared to *rbcl* due to its specific level of accuracy at species level (Yu *et al*. 2011). Maturation of K (*matK*) is a gene present in the chloroplast and is located between exons 5′ and 3′ of *trnK* and lysine-tRNA (Enan & Ahmed 2014). The *matK* gene has been widely used as barcode in *angiospermae* plants (Yu *et al*. 2011). In the present paper, we use the cpDNA *maturase K* marker to identify *Z. acanthopodium* and evaluated its use as DNA barcoding marker.

## MATERIALS AND METHODS

### Plant Samples

**Figure 1:**
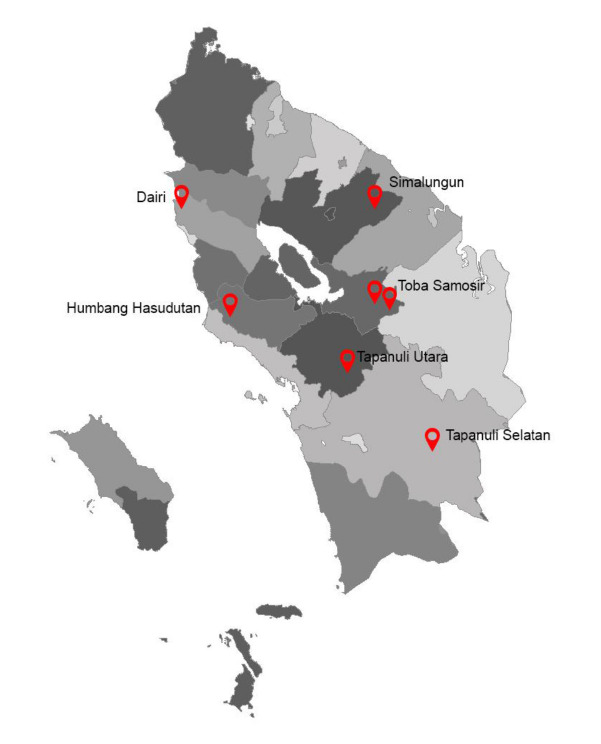
Sampling locations in North Sumatra, Indonesia at seven points in six regencies. (*Source*: Google Map and processed using ArcGis software).

*Z. acanthopodium* plant sample is obtained from various regions in North Sumatra province which are represented by six regions namely South Tapanuli, North Tapanuli, Humbang Hasudutan, Dairi, Simalungun and Toba Samosir (Fig. 1). The samples used in this research are fresh leaf samples obtained by field exploration. The outgroup used to compare the barcoding sequences obtained were *Citrus x paradisi* and *Melicope glabra*, while the ingroup used were several species from the genus *Zanthoxylum* (Table 1).

### DNA Isolation and Amplification of cDNA *matK* sequence

The DNA isolation was carried out by following the procedure of the GeneJet Plant Genomic DNA Purification Kit (Thermo Fisher Scientific, Waltham, MA, USA). A total of 100 mg of *Z. acanthopodium* leaf sample was crushed with an additional Lysis Buffer A of 350 μL. The solution is homogenised with the addition of 50 μL Lysis Buffer B and 20 μL of RNAse A and incubated at 65°C for 10 min. Subsequently, the solution was added with 130 μL of precipitation solution and centrifuged to isolate the supernatant. The supernatant in turn was added with 400 μL of Plant gDNA Binding Solution and 400 μL of 96% ethanol. The solution is then transferred into a purification column and centrifuged at 8,000 rpm for 1 min. The purification process was carried out using Wash Buffer I and Wash Buffer II in the purification column. The genomic DNA elution was performed with the addition of 100 μL of Elution Buffer, followed by centrifugation at 10,000 rpm for 1 min. Purified DNA is then stored at −20°C.

The *matK* sequence is amplified using matK-F 5′-ACC CAG TCC ATC TGG AAA TCT TGG TTC-3′ and matK-R 5′-CGT ACA GTA CTT TTG TGT TTA CGA G-3′ primers (Ki-Joong Kim, School of Life Sciences and Biotechnology, Korea University, Korea, unpublished) with total reaction volume of 25 μL [2.5 μL of DNA template; 2.5 μL of matK-F primer, 2.5 μL of matK-R primer; 5 μL of distilled water, and 12.5 μL of PCR mix (MyTaq HS Red Mix (Bioline, USA))] mixture with a final concentration of template DNA of 50 ng. Amplification of cpDNA *matK* sequence with a predenaturation condition of 5 min at 97°C, followed by 40 cycles under denaturation reaction conditions at 94°C for 5 min, annealing at 52°C, and extension at 72°C for 1 min, then the PCR process terminated with post-extension at 72°C for 5 min. PCR products were visualised using agarose gel 1% plus 5 μL of SYBR ® Safe DNA Gel Stain (Invitrogen, USA). Results PCR products that show positive results (DNA bands are clearly visible) will be sent to the First Base DNA Sequencing Service in Singapore for sequencing.

### Barcoding and Phylogenetic Analysis

The result of *matK* sequencing was analysed using Bioedit 7.0.1 (Hall 1999) program to determine consensus sequence. Phylogenetic tree, nucleotide composition, and genetic distance were carried out using MEGA (Molecular Evolutionary Genetic Analysis) version 7 (Kumar *et al*. 2016) program based on alignment of sequence data. The method used for analysis is Maximum Likelihood with 1,000 bootstrap replicates.

## RESULTS

**Figure 2:**
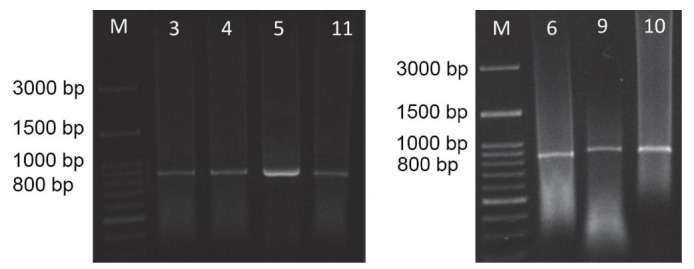
Visualisation of PCR results of *matK* gene sequences with agarose: (3) South Tapanuli; (4) North Tapanuli; (5) Humbang Hasudutan; (11) Toba Samosir 1; (6) Dairi; (9) Simalungun; (10) Toba Samosir 2.

Amplification of *matK* gene from the chloroplast of *Z. acanthopodium* genome was successful. The result of PCR visualised using agarose shows a single band, which means that the *matK* sequence has been successfully amplified (Fig. 2).

The result of *matK* gene sequence alignment shows that the *matK* gene sequence in *Z. acanthopodium* consists of 850 characters. Based on the data, 827 of the characters are conservative sequences, two are potentially informative parsimony characters, and five are variable sites. The result of alignment showed that the *matK* gene sequence on *Z. acanthopodium* had very high homology level (97.3%). The base frequency of the *matK* gene sequence on *Z. acanthopodium* is 35.16% (T), 19.11% (C), 27.34% (A), and 18.35% (G). This sequence is rich in T/A (62.47%), whereas G/C content is 37.48% (Table 2).

**Figure 3:**
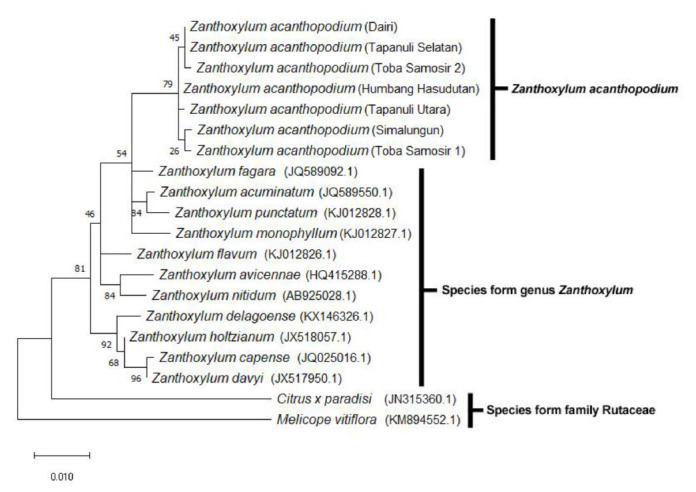
The phylogenetic tree of *matK* sequence from Z. *acanthopodium* and *outgroup* (species in the *Zanthoxylum* genus and Rutaceae family) as a result of reconstruction using the Maximum Likelihood method based on the kimura-2-parameter model. Branching is analysed with a bootstrap value of 1000x.

The phylogenetic tree presented in Fig. 3 was constructed using Maximum Likelihood and 1000x bootstrap methods. This method is used to identify differences in genetic distance and analyse similarity between samples.

There are total of seven accessions of *Z. acanthopodium* clustered on the same branch and separated from other species of the *Zanthoxylum* genus and those from the Rutaceae family. Analysis of phylogenetic trees based on the *matK* sequence shows that the *Zanthoxylum* genus originated from one common ancestor. The results of this analysis also show that the *matK* sequence can be used as DNA Barcoding on *Z. acanthopodium*. It also indicates that the genetic distance between *Z. acanthopodium* species is very low compared to that between species in the *Zanthoxylum* genus or Rutaceae family. The lowest genetic distance between *Z. acanthopodium* is found in *Z. acanthopodium* from Dairi, Tapanuli Selatan and Toba Samosir 2. The genetic distance between the lowest *Zanthoxylum* genus is between *Z. capense* and *Z. davyi* (0.001) while the highest genetic distance is between *Z. nitidum* with *Z. capense* (0.023). Outgroup species from the Rutaceae family other than the *Zanthoxylum* genus showed significant differences (see Table 3).

## DISCUSSION

Various molecular markers have been developed for the purpose of identification of plant species. One of the most recommended molecular markers for identification purposes is DNA barcoding. One of the gene sequences contained chloroplast DNA, the *matK* is a common barcode used in species identification. The *matK* gene is recommended by *The Consortium for the Barcode of Life* (Hollingworth *et al*. 2009).

Andaliman (*Z. acanthopodium* DC) is a commonly found plant in the northern part of Sumatra and has an important role in the customs and culture of Batak tribe. The fruit of this plant is used in a variety of traditional Batak cuisines in the North Sumatra province, Indonesia (Kristanty & Suriawati 2015). Research on this plant focuses on secondary metabolites with benefits in health and food (Li *et al*. 2012).

The *matK-F* and *matK-R* primers used in this study successfully amplified the *matK* gene sequence at a length of 850 bp. Previous study indicated that this primer can amplify various species of *Angiospermae* trees at amplification length of 830 bp–857 bp (Tosh *et al*. 2016). This primer has also successfully amplified the genus *Abelmochus* (Fattah *et al*. 2014), *Ficus* (Moraceae) (Li *et al*. 2012), *Sycygium* (Tallei *et al*. 2016), Lilianaceae (Ma *et al*. 2014) and Vitaceae (Habib *et al*. 2017). The results showed that the sequence of *matK* gene on *Z. acanthopodium* had high homology level (97.3%). This value is higher than the homology level of 14 species of Anacardiaceae in the ITS core genome area of 75% (Hidayat *et al*. 2011), *trnL-F* sequence on *Bouea macrophylla* (97.26%) (Harsono *et al*. 2017), and sequences of *matK* genes in species within the Fabaceae family (96%) (Gao *et al*. 2011). This value is lower than the homology level of *trnL-F* gene sequence on *Bouea oppositifolia* (97.48%) (Harsono *et al*. 2017).

Table 3 shows that the genetic distance in the *matK gene* sequence on *Z. acanthopodium* is very small (<1%). This shows that the *matK gene* sequence has very high conserved. Variation of order of sequences in cpDNA are generally caused by a single nucleotide mutation that has occurred over a very long period (Fitmawati & Hartana 2010; Borsch *et al*. 2003). Changes in the nucleotide sequence in the chloroplast genome are able to provide important information about the species evolution because the chloroplast genome is inherited maternally where small changes take place over a very long period of time (Hancock 2003), whereas in the DNA core changes occur due to recombination of both parentals.

The result of phylogenetic tree analysis in Fig. 3 shows that phylogenetic tree produced is monophyletic with three main groups. The first group is those of *Z. acanthopodium* species consisting of seven accessions. The second group consists of species belonging to the genus *Zanthoxylum* in addition to *acanthopodium* species. The third group is an outgroup group that all species in the *Zanthoxylum* genus derived from *Citrus x paradisi* and *Melicope vitiflora* belonging to the Rutaceae family. This is consistent with the statement of Taberlet *et al*. (1991) which states that the chloroplast genome is best used for inter-species kinship analysis but is less suitable in interspecies kinship analysis.

The variety indicated by cpDNA markers is relatively different from the diversity shown by morphological markers. The patterns emerging from cpDNA markers do not necessarily correlate with patterns generated from morphological markers, and vice versa. This is possible because the expression at the morphological level is the result of recombination of two parents and environmental factors. In addition, the gene sequences located on chloroplast DNA experience a lower rate of evolution than that of core DNA (Taberlet *et al*. 1991). The noncoding area has a high mutation rate, making the variations appear more and more informative when compared with the coding area (Taberlet *et al*. 1991; Hamilton MB. (1999).

## CONCLUSION

Based on the results of research in this study, it can be concluded that the cpDNA *matK* marker can be amplified by the length of 850 bp on *Z. acanthopodium*. cpDNA *matK* marker can be used as DNA barcoding to identify *Z. acanthopodium*. cpDNA *matK* markers can also be used to separate *Z. acanthopodium* from other species of the genus *Zanthoxylum* and separate from outgroups of the Rutaceae family (*Citrus x paradisi* and *Melicope vitiflora*).

## Figures and Tables

**Table 1:** Sample of *Z. acanthopodium* and *outgroup* species of the *Zanthoxylum* genus and from Rutaceae family.

No	Species	Accession Number	Origin
1	*Zanthoxylum holtzianum*	JX518057.1	South Africa
	(outgroup)		
2	*Z. capense* (outgroup)	JQ025016.1	South Africa
3	*Z. davyi* (outgroup)	JX517950.1	South Africa
4	*Z. avicennae* (outgroup)	HQ415288.1	China
5	*Z. nitidum* (outgroup)	AB925028.1	Cambodia
6	*Z. fagara* (outgroup)	JQ589092.1	Costa Rica
7	*Z. acuminatum* (outgroup)	JQ589550.1	Costa Rica
8	*Z. delagoense* (outgroup)	KX146326.1	Mozambique
9	*Z. punctatum* (outgroup)	KJ012828.1	Puerto Rico
10	*Z. monophyllum*(outgroup)	KJ012827.1	Puerto Rico
11	*Z. flavum* (outgroup)	KJ012826.1	Puerto Rico
12	*Citrus x paradisi* (outgroup)	JN315360.1	India
13	*Melicope glabra* (outgroup)	KJ709002.1	Singapore
14	*Z. acanthopodium*	Sample	Indonesia
15	*Z. acanthopodium*	Sample	Indonesia
16	*Z. acanthopodium*	Sample	Indonesia
17	*Z. acanthopodium*	Sample	Indonesia
18	*Z. acanthopodium*	Sample	Indonesia
19	*Z. acanthopodium*	Sample	Indonesia
20	*Z. acanthopodium*	Sample	Indonesia

**Table 2:** Composition of nucleotides, A/T content, and G/C content in the *matK* gene sequence on *Z. acanthopodium*.

Species	Composition (%)				Total	Content (%)	
	
	T(U)	C	A	G		A/T	G/C
*Z. acanthopodium* (Humbang Hasudutan)	34.9	19.4	27.1	18.6	850	62	38
*Z. acanthopodium* (Dairi)	35.4	19.2	27.1	18.3	804	62.5	37.5
*Z. acanthopodium* (Simalungun)	34.8	18.7	28.1	18.4	787	62.9	37.1
*Z. acanthopodium* (Tapanuli Selatan)	35.4	19.2	27	18.4	808	62.4	37.6
*Z. acanthopodium* (Tapanuli Utara)	35.1	19.3	27.2	18.4	794	62.3	37.7
*Z. acanthopodium* (Toba Samosir 1)	35.3	19.1	27.4	18.1	827	62.7	37.2
*Z. acanthopodium* (Toba Samosir 2)	35.2	18.9	27.5	18.3	829	62.7	37.2

Average	35.16	19.11	27.34	18.36	814.14	62.5	37.45

**Table 3:** Genetic distance of Andaliman (*Z. acanthopodium*) with species in *Zanthoxylum* genus and Rutaceae family.

No	Species	1	2	3	4	5	6	7	8	9	10
1	*Citrus x paradisi* (JN315360.1)	0									
2	*Melicope vitiflora* (KM894552.1)	0.095	0								
3	*Z. acuminatum* (JQ589550.1)	0.054	0.066	0							
4	*Z. avicennae* (HQ415288.1)	0.054	0.068	0.012	0						
5	*Z. capense* (JQ025016.1)	0.057	0.070	0.018	0.021	0					
6	*Z. davyi* (JX517950.1)	0.055	0.071	0.016	0.019	0.001	0				
7	*Z. delagoense* (KX146326.1)	0.052	0.063	0.013	0.016	0.010	0.009	0			
8	*Z. fagara* (JQ589092.1)	0.055	0.068	0.007	0.013	0.019	0.018	0.015	0		
9	*Z. flavum* (KJ012826.1)	0.055	0.068	0.010	0.013	0.019	0.018	0.015	0.012	0	
10	*Z. holtzianum* (JX518057.1)	0.050	0.067	0.012	0.015	0.006	0.004	0.004	0.013	0.013	0
11	*Z. monophyllum* (KJ012827.1)	0.055	0.066	0.010	0.016	0.022	0.021	0.018	0.012	0.012	0.016
12	*Z. nitidum* (AB925028.1)	0.055	0.071	0.013	0.010	0.023	0.021	0.018	0.015	0.015	0.016
13	*Z. punctatum* (KJ012828.1)	0.054	0.070	0.004	0.016	0.022	0.021	0.018	0.012	0.015	0.016
14	*Z. acanthopodium* (Humbang Hasudutan)	0.055	0.068	0.007	0.013	0.018	0.016	0.015	0.009	0.012	0.013
15	*Z. acanthopodium* (Dairi)	0.054	0.067	0.009	0.015	0.016	0.015	0.013	0.010	0.013	0.012
16	*Z. acanthopodium* (Simalungun)	0.054	0.070	0.009	0.015	0.016	0.015	0.013	0.010	0.013	0.012
17	*Z. acanthopodium* (Tapanuli Selatan)	0.054	0.067	0.009	0.015	0.016	0.015	0.013	0.010	0.013	0.012
18	*Z. acanthopodium* (Tapanuli Utara)	0.057	0.070	0.009	0.015	0.016	0.015	0.016	0.010	0.013	0.015
19	*Z. acanthopodium* (Toba Samosir 1)	0.057	0.070	0.009	0.012	0.019	0.018	0.016	0.010	0.013	0.015
20	*Z. acanthopodium* (Toba Samosir 2)	0.054	0.067	0.009	0.015	0.016	0.015	0.013	0.010	0.013	0.012

No	Species	11	12	13	14	15	16	17	18	19	20
11	*Z. monophyllum* (KJ012827.1)	0									
12	*Z. nitidum* (AB925028.1)	0.018	0								
13	*Z. punctatum* (KJ012828.1)	0.015	0.018	0							
14	*Z. acanthopodium* (Humbang Hasudutan)	0.012	0.015	0.012	0						
15	*Z. acanthopodium* (Dairi)	0.013	0.016	0.013	0.001	0					
16	*Z. acanthopodium* (Simalungun)	0.013	0.016	0.013	0.001	0.003	0				
17	*Z. acanthopodium* (Tapanuli Selatan)	0.013	0.016	0.013	0.001	0.000	0.003	0			
18	*Z. acanthopodium* (Tapanuli Utara)	0.013	0.016	0.013	0.001	0.003	0.003	0.003	0		
19	*Z. acanthopodium* (Toba Samosir 1)	0.013	0.013	0.013	0.001	0.003	0.003	0.003	0.003	0	
20	*Z. acanthopodium* (Toba Samosir 2)	0.013	0.016	0.013	0.001	0.000	0.003	0.000	0.003	0.003	0
